# A hierarchical model for clustering m^6^A methylation peaks in MeRIP-seq data

**DOI:** 10.1186/s12864-016-2913-x

**Published:** 2016-08-22

**Authors:** Xiaodong Cui, Jia Meng, Shaowu Zhang, Manjeet K. Rao, Yidong Chen, Yufei Huang

**Affiliations:** 1 Department of Electrical and Computer Engineering, University of Texas, San Antonio, TX 78249 USA; 2 Department of Biological Science, Xi’an Jiaotong-liverpool University, Suzhou, 215123 China; 3 College of Automation, Northwestern Polytechnical University, Xi’an, 710072 China; 4 Depeartment of Epidemiology and Biostatistics, University of Texas Health Science Center, San Antonio, TX 78229 USA; 5 Greehey Children’s Cancer Research Institute, University of Texas Health Science Center, San Antonio, TX 78229 USA

## Abstract

**Background:**

The recent advent of the state-of-art high throughput sequencing technology, known as Methylated RNA Immunoprecipitation combined with RNA sequencing (MeRIP-seq) revolutionizes the area of mRNA epigenetics and enables the biologists and biomedical researchers to have a global view of *N*^6^-Methyladenosine (m^6^A) on transcriptome. Yet there is a significant need for new computation tools for processing and analysing MeRIP-Seq data to gain a further insight into the function and m^6^A mRNA methylation.

**Results:**

We developed a novel algorithm and an open source R package (http://compgenomics.utsa.edu/metcluster) for uncovering the potential types of m^6^A methylation by clustering the degree of m^6^A methylation peaks in MeRIP-Seq data. This algorithm utilizes a hierarchical graphical model to model the reads account variance and the underlying clusters of the methylation peaks. Rigorous statistical inference is performed to estimate the model parameter and detect the number of clusters. MeTCluster is evaluated on both simulated and real MeRIP-seq datasets and the results demonstrate its high accuracy in characterizing the clusters of methylation peaks. Our algorithm was applied to two different sets of real MeRIP-seq datasets and reveals a novel pattern that methylation peaks with less peak enrichment tend to clustered in the 5′ end of both in both mRNAs and lncRNAs, whereas those with higher peak enrichment are more likely to be distributed in CDS and towards the 3′end of mRNAs and lncRNAs. This result might suggest that m^6^A’s functions could be location specific.

**Conclusions:**

In this paper, a novel hierarchical graphical model based algorithm was developed for clustering the enrichment of methylation peaks in MeRIP-seq data. MeTCluster is written in R and is publicly available.

**Electronic supplementary material:**

The online version of this article (doi:10.1186/s12864-016-2913-x) contains supplementary material, which is available to authorized users.

## Background

*N*^*6*^-methyl-adenosine (m^6^A) is the most abundant modification among 100 types of identified RNA modifications in eukaryotic mRNA/lncRNA [1, 2]. Even though m^6^A was found existing in mammalian mRNAs in as early as 1970s [3], its biological relevance remains unclear due to the difficulties in identifying global m^6^A sites in mRNA [4]. In 2013, the m^6^A demethylase Fat mass and obesity associated protein (FTO) was first discovered [5], to be able to reverse the m^6^A modification in mRNA and it revived our interests of studying m^6^A in mRNA. To date, ALKBH5 is identified as another demethylase [6] and the methyltransferase like 3/14 (METTL3/METTL14) and Wilms’ tumor 1-assoicating protein (WTAP) are discovered to be subunits of the m^6^A methyltransferase complex [7, 8]. All these findings provide strong evidences to show that m^6^A is a dynamic modification and suggest that it may play a critical role in exerting post-transcriptional functions in mRNA metabolism [9–11].

These new wave of breakthroughs cannot be achieved without the recent development of MeRIP-seq [12, 13], which was successfully developed to reveal the transcriptome-wide distribution of m^6^A in human and mouse cells. In this essay, mRNA is first chemically fragmented into approximately 100-nucleotide (nt) long before immunoprecipitation with anti-m^6^A antibody. Then, the immunoprecipitated (IPed) methylated mRNA fragments and the un-immunoprecipited input control mRNA fragments are subjected to high-throughput sequencing [14]. The sequenced IP and input reads are aligned to the transcriptome and reads enrichment of IP out of the combined reads in IP and input samples are examined to predict to loci of methylation sites and infer the degree of methylation. We have previously developed exomePeak [15, 16] and HEPeak [17], two algorithms for detecting m^6^A peaks in MeRIP-seq. Although MeRIP-seq and subsequent computational peak-calling analysis provide an accurate landscape of m^6^A methylation in transcriptome, the complete mechanisms of this methylation still remains unclear. Just like gene expression where co-expression might suggest co-regulation or similar gene functions, sites with similar methylation degree could be related to similar methylation mechanisms. Therefore, there is a need to develop algorithms to uncover co-methylation pattern in MeRIP-seq data. In this paper, we model the methylation degrees of m^6^A peaks as a mixture of the Beta-binomial distributions and propose an expectation-maximization based clustering algorithm to uncover the co-methylation patterns.

## Methods

In this section, we first describe the proposed generative model to define m^6^A peak clusters and then derive the Expectation-Maximization algorithm for the inference. In the end, we discuss a Bayesian Information Criterion (BIC) [18] for selecting the optimal number of m^6^A peak clusters.

### The proposed graphical model for clustering RNA methylation peaks

**Fig. 1:**
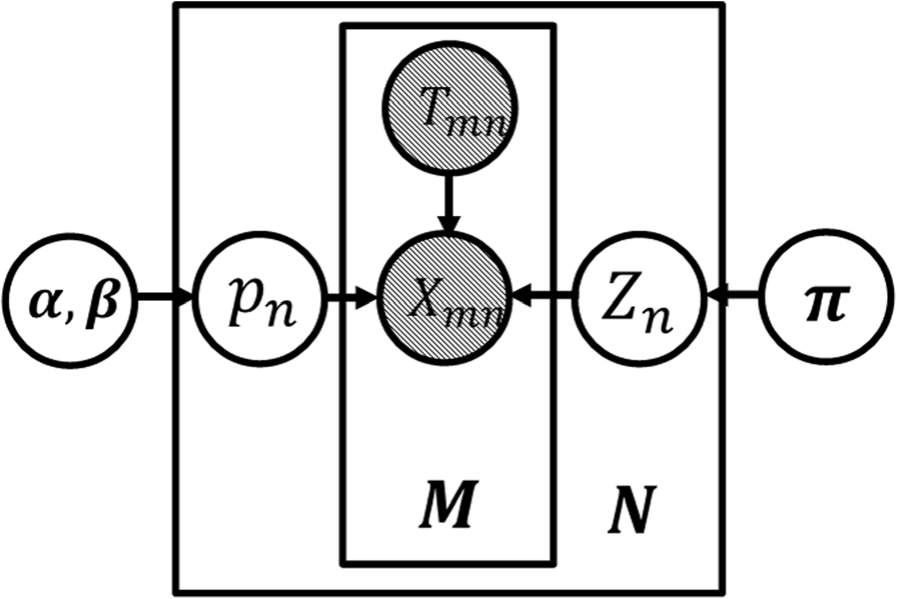
The proposed graphical model for peak clusters

The proposed graphical model for clustering of m^6^A peaks in MeRIP-seq data is shown in Fig. 1. Suppose we have identified a set of *N* m^6^A peaks, by using peak-calling software such as exomePeak or HEPeak. The goal is to cluster these peaks according to their methylation degree, which is defined as IP reads count divided by the total count of IP and control reads. For the *n*_*th*_ m^6^A peak, let *Z*_*n*_ ∈ {1, 2,.., *K*} denote the index of the particular methylation cluster that *n*-th peak belongs to, with *K* representing the total number of clusters, then *Z*_*n*_ follows a discrete distribution

1 $$ P\left({Z}_n\Big|\boldsymbol{\uppi} \right)={\displaystyle \prod_{k=1}^K{\pi_k}^{I\left({Z}_n=k\right)}} $$

where *π*_*k*_ is the unknown probability that an m^6^A peak belongs to cluster *k*, where ∑_*K*_*π*_*k*_ = 1 and *I*(⋅) is the indicator function. Also, let the observed reads count in the *n*_*th*_ peak of the *m*_*th*_ IP replicate sample be *X*_*m*,*n*_ and that of the *m*_*th*_ input replicate denote as *Y*_*mn*_. Under the assumption that reads count follows a Poisson distribution, the reads count *X*_*mn*_ given the total reads account *T*_*mn*_ = *X*_*mn*_ + *Y*_*mn*_ can be shown to follow a Binomial distribution

2 $$ P\left({X_m}_n\Big|{p}_n,{Z}_n\right)={{\displaystyle \prod_{k=1}^K\left(\left(\begin{array}{c}\hfill {T}_{mn}\hfill \\ {}\hfill {X}_{mn}\hfill \end{array}\right){p_n}^{X_{mn}}{\left(1-{p}_n\right)}^{Y_{mn}}\right)}}^{I\left({Z}_n=k\right)} $$

where is *p*_*n*_ represents unknown methylation degree at the *n*_*th*_ Peak of the *m*_*th*_ replicate. In order to model the variance of the replicates for the *n*_*th*_ peak, given cluster assignment *Z*_*n*_, *p*_*n*_ is assumed to follow the Beta distribution

3 $$ P\left(p\Big|{Z}_n\right)={\displaystyle \prod_{k=1}^K Beta{\left({\alpha}_k,{\beta}_k\right)}^{I\left({Z}_n=k\right)}} $$

Therefore, after integrating the variable ${p}_n$, ${X}_{mn}$ follows a mixture of Beta-binomial distribution

4 $$ \begin{array}{l}P\left({X}_{mn}\Big|{Z}_n;\boldsymbol{\upalpha}, \boldsymbol{\upbeta} \right)={\displaystyle \sum_{p_n}P\left({X_m}_n\Big|{p}_n,{Z}_n\right)P\left({p}_n\Big|\boldsymbol{\upalpha}, \boldsymbol{\upbeta} \right)}\\ {}={{\displaystyle \prod_{k=1}^K\left(C\bullet \frac{\varGamma \left({X}_{mn}+{\alpha}_k\right)\varGamma \left({Y}_{mn}+{\beta}_k\right)}{\varGamma \left({T}_{mn}+{\alpha}_k+{\beta}_k\right)}\frac{\varGamma \left({\alpha}_k+{\beta}_k\right)}{\varGamma \left({\alpha}_k\right)\varGamma \left({\beta}_k\right)}\right)}}^{I\left\{{Z}_n=k\right\}}\end{array} $$

where *α* = [*α*_1_, *α*_2_,.., *α*_*K*_]^*T*^, ***β*** = [*β*_1_, *β*_2_,.., *β*_*K*_]^*T*^ are the unknown parameters of Beta distribution and *C* is the normalization constant. Thus, by considering the *N* m^6^A peaks in *M* replicates, the joint distribution is

5 $$ P\left(\mathbf{X},\mathbf{Z}\Big|\boldsymbol{\upalpha}, \boldsymbol{\upbeta}, \boldsymbol{\uppi} \right)={\displaystyle \prod_{n=1}^N{\displaystyle \prod_{m=1}^M{\displaystyle \prod_{k=1}^K{\left({\pi}_kBB\left({X}_{mn}\Big|{Z}_n\right)\right)}^{I\left\{{Z}_n=k\right\}}}}} $$

where *BB*(*X*_*mn*_|*Z*_*n*_) represents formula (3). Then, the log-likelihood of the observed data can be expressed as

6 $$ \begin{array}{l}l= \lg P\left(\mathbf{X}\Big|\boldsymbol{\upalpha}, \boldsymbol{\upbeta}, \boldsymbol{\uppi} \right)= \lg {\displaystyle \sum_{\mathbf{Z}}P\left(\mathbf{X},\mathbf{Z}\Big|\boldsymbol{\upalpha}, \boldsymbol{\upbeta}, \boldsymbol{\uppi} \right)}\\ {}={\displaystyle \sum_{n=1}^N{\displaystyle \sum_{m=1}^M \lg {\displaystyle \sum_{k=1}^K{\pi}_kBB\left({X}_{mn}\Big|{Z}_n;\boldsymbol{\upalpha}, \boldsymbol{\upbeta} \right)}}}\end{array} $$

where ***Z*** = [*Z*_1_, *Z*_2_, …, *Z*_*N*_]^*T*^, **X** = [***X***_***1***,_^***T***^***X***_***2***,…,_^***T***^***X***_***N***_^***T***^]^***T***^ and ${\boldsymbol{X}}_{\boldsymbol{n}}={\left[{X}_{1n},{X}_{2n},\dots, {X}_{Mn}\right]}^T$. The goal of inference is to predict the cluster index ${Z}_n$ for all the peaks and estimate the unknown model parameters $\boldsymbol{\theta} =\left[\boldsymbol{\alpha}, \boldsymbol{\beta}, \boldsymbol{\pi} \right]$. Next, we first discuss the maximum likelihood solution for parameter inference, based which an EM algorithm is introduced afterwards to perform model parameters inference and cluster assignment jointly.

### Parameter inference by the Newton’s method

Given that the cluster indices are known, the model parameters can be inferred by the maximum likelihood criterion as

7 $$ {\widehat{\uptheta}}_{ML}=\underset{\boldsymbol{\uptheta}}{ \arg \max (l)}. $$

Given (5–6), the log-likelihood $l$ can be rewritten as

8 $$ \begin{array}{l}l={\displaystyle \sum_{n=1}^N{\displaystyle \sum_{m=1}^M \lg {\displaystyle \sum_{k=1}^Kq\left({Z}_{nk}\right)\frac{\pi_kBB\left({X}_{mn}\Big|{Z}_n\right)}{q\left({Z}_{nk}\right)}}}}\\ {}\ge {\displaystyle \sum_{n=1}^N{\displaystyle \sum_{m=1}^M{\displaystyle \sum_{k=1}^Kq\left({Z}_{nk}\right)\left[ \lg {\pi}_k+ \lg BB\left({X}_{mn}\Big|{Z}_n\right)- \lg q\left({Z}_n\right)\right]}}}\\ {}={\displaystyle \sum_{n=1}^N{\displaystyle \sum_{k=1}^KM\cdotp q\left({Z}_{nk}\right) \lg {\pi}_k}}-{\displaystyle \sum_{n=1}^N{\displaystyle \sum_{k=1}^KM\cdotp q\left({Z}_{nk}\right) \lg q\left({Z}_{nk}\right)}}\\ {}+{\displaystyle \sum_{n=1}^N{\displaystyle \sum_{m=1}^M{\displaystyle \sum_{k=1}^Kq\left({Z}_{nk}\right)\left[\begin{array}{l}\varPhi \left({\alpha}_k+{\beta}_k\right)-\varPhi \left({T}_{mn}+{\alpha}_k+{\beta}_k\right)+\varPhi \left({X}_{mn}+{\alpha}_k\right)\\ {}+\varPhi \left({Y}_{mn}+{\beta}_k\right)-\varPhi \left({\alpha}_k\right)-\varPhi \left({\beta}_k\right)\end{array}\right]}}}\end{array} $$

where $\Phi = \lg \Gamma \left(\cdotp \right)$ and $q\left({Z}_n\right)=P\left({Z}_n=k|\boldsymbol{X}\right)$. Here, given that $q\left({Z}_n\right)$ is a complex simplex, according to the Jensen’s inequality, the lower bound of $l$ is achieved when $q\left({Z}_n\right)=P\left({Z}_n|\boldsymbol{X}\right)$. With a little abuse of notation, $l$ denotes the lower bound of (7). Given the equality constrain ${\sum}_K{\pi}_k=1$, the parameters of $\boldsymbol{\pi}$ can be computed by maximizing $l$ and its dual problem with Lagrange multiplier $\lambda$ is

9 $$ \max \kern1em g\left(\boldsymbol{\uppi}, \lambda \right)={\displaystyle \sum_n{\displaystyle \sum_m{\displaystyle \sum_kq\left({Z}_n\right) \lg {\pi}_k}+\lambda \left({\displaystyle \sum_k{\pi}_k-1}\right)}} $$

then $\lambda$ and $\pi$ can be calculated as

10 $$ \begin{array}{l}\lambda =-N\cdotp M\\ {}{\pi}_k=\frac{1}{N}{\displaystyle \sum_{n=1}^NP\left({Z}_n=k\Big|{\mathbf{X}}_{\mathbf{n}}\right)}\end{array} $$

Due to lack of analytical solution for the derivatives of $l$ with respect to $\boldsymbol{\alpha}$ and $\boldsymbol{\beta}$, a Newton’s method is applied and the the gradient can be computed as

11 $$ {J}^k=\left[\begin{array}{c}\hfill {\displaystyle \sum_{n=1}^Nq\left({Z}_{nk}\right)\left[\begin{array}{l}\varPhi \left({\alpha}_k+{\beta}_k\right)\cdotp M-{\displaystyle \sum_{m=1}^M\varPhi \left({T}_{mn}+{\alpha}_k+{\beta}_k\right)}\\ {}-\varPhi \left({\alpha}_k\right)\cdotp M+{\displaystyle \sum_{m=1}^M\varPhi \left({X}_{mn}+{\alpha}_k\right)}\end{array}\right]}\hfill \\ {}\hfill {\displaystyle \sum_{n=1}^Nq\left({Z}_{nk}\right)\left[\begin{array}{l}\varPhi \left({\alpha}_k+{\beta}_k\right)\cdotp M-{\displaystyle \sum_{m=1}^M\varPhi \left({T}_{mn}+{\alpha}_k+{\beta}_k\right)}\\ {}-\varPhi \left({\beta}_k\right)\cdotp M+{\displaystyle \sum_{m=1}^M\varPhi \left({Y}_{mn}+{\beta}_k\right)}\end{array}\right]}\hfill \end{array}\right] $$

and the Hessian is

12 $$ \begin{array}{l}{H^k}_{1,1}={\displaystyle \sum_{n=1}^Nq\left({Z}_{nk}\right)\left[\begin{array}{l}{\varPhi}^{\hbox{'}}\left({\alpha}_k+{\beta}_k\right)\cdotp M-{\displaystyle \sum_{m=1}^M{\varPhi}^{\hbox{'}}\left({T}_{mn}+{\alpha}_k+{\beta}_k\right)}\\ {}-{\varPhi}^{\hbox{'}}\left({\alpha}_k\right)\cdotp M+{\displaystyle \sum_{m=1}^M{\varPhi}^{\hbox{'}}\left({X}_{mn}+{\alpha}_k\right)}\end{array}\right]}\\ {}{H^k}_{2,2}={\displaystyle \sum_{n=1}^Nq\left({Z}_{nk}\right)\left[\begin{array}{l}{\varPhi}^{\hbox{'}}\left({\alpha}_k+{\beta}_k\right)\cdotp M-{\displaystyle \sum_{m=1}^M{\varPhi}^{\hbox{'}}\left({T}_{mn}+{\alpha}_k+{\beta}_k\right)}\\ {}-{\varPhi}^{\hbox{'}}\left({\beta}_k\right)\cdotp M+{\displaystyle \sum_{m=1}^M{\varPhi}^{\hbox{'}}\left({Y}_{mn}+{\beta}_k\right)}\end{array}\right]}\\ {}{H^k}_{1,2}={H^k}_{2,1}\\ {}={\displaystyle \sum_{n=1}^Nq\left({Z}_{nk}\right)\left[{\phi}^{\hbox{'}}\left({\alpha}_k+{\beta}_k\right)\cdotp M-{\displaystyle \sum_{m=1}^M{\phi}^{\hbox{'}}\left({T}_{mn}+{\alpha}_k+{\beta}_k\right)}\right]}.\end{array} $$

Then, the parameters for the ${k}_{th}$ cluster can be updated iteratively as

13 $$ \left[\begin{array}{c}\hfill {\alpha^k}_{new}\hfill \\ {}\hfill {\beta^k}_{new}\hfill \end{array}\right]=\left[\begin{array}{c}\hfill {\alpha^k}_{old}\hfill \\ {}\hfill {\beta^k}_{old}\hfill \end{array}\right]-{\left({H}^k\right)}^{-1}{J}^k $$

### m^6^A peak cluster assignment

Assigning m^6^A peak to a cluster amounts to inferring cluster index ${Z}_n$, whose posterior probability given $\boldsymbol{\theta}$ can be written as

14 $$ \begin{array}{l}P\left({Z}_n=k\Big|{\mathbf{X}}_{\mathbf{n}},\boldsymbol{\uptheta} \right)=\frac{P\left({Z}_n=k,{\mathbf{X}}_{\mathbf{n}}\Big|\boldsymbol{\uptheta} \right)}{{\displaystyle \sum_{k=1}^KP\left({Z}_n=k,{\mathbf{X}}_{\mathbf{n}}\Big|\boldsymbol{\uptheta} \right)}}\\ {}=\frac{\pi_k\cdotp {\displaystyle \prod_{m=1}^MBB\left({X}_{mn}\Big|{Z}_n=k,\boldsymbol{\uptheta} \right)}}{{\displaystyle \sum_{k=1}^K{\pi}_k{\displaystyle \prod_{m=1}^MBB\left({X}_{mn}\Big|{Z}_n=k,\boldsymbol{\uptheta} \right)}}}.\end{array} $$

However, $P\left({Z}_n=k|{\boldsymbol{X}}_{\boldsymbol{n}},\boldsymbol{\theta} \right)$ cannot be computed directly, because parameter $\boldsymbol{\theta}$ is also unknown. To circumvent the difficulty, we developed an EM [19] algorithm to infer ${Z}_n$ and estimate the model parameters $\boldsymbol{\theta}$ in an iterative fashion. The steps of the proposed EM algorithm are described in the following

Repeat until convergence achieved:

E-step: use the previous computed parameters ${\boldsymbol{\theta}}_{\boldsymbol{old}}$ to update the posterior probability of the hidden states $P\left({Z}_n=k|{\boldsymbol{X}}_{\boldsymbol{n}},\boldsymbol{\theta} \right)$ according to (13).

M-step: maximize the lower bound $l$ in (7) and estimate parameters ${\boldsymbol{\theta}}_{\boldsymbol{new}}$ according to (12).

### Selection of the number of states by Bayesian information criterion (BIC)

Note that the total number of states *K* is also unknown. In order to determine *K*, the BIC is applied search in the range of 2 to 15. The best number of states is selected by the lowest BIC, which is denoted as

15 $$ BIC=-2\widehat{l}+2K \lg N $$

where $\widehat{l}$ is the estimated log-likelihood when the EM algorithm converges.

## Results

### Performance evaluation by simulation

The performance was evaluated by simulation where the true states of methylation peaks are known. Each peak was simulated independently, where the reads count was generated according to the proposed graphical model in Fig. 1. Notably, from (3), we can determine that the distribution of the methylation degree follows the following mixture Beta distribution

16 $$ P(p)={\displaystyle \sum_{k=1}^K{\pi}_k Beta\left({\alpha}_k,{\beta}_k\right)} $$

where the *k*th Beta distribution model the methylation degree in cluster *k*. In our case, we assume there are $K=4$ clusters and $\boldsymbol{\pi} =\left[0.3,0.4,0.2,0.1\right]$. Note that the degree *p* may vary vastly when the variance of the Beta distribution is large. In addition, the total reads count ${T}_n$ of the ${n}_{th}$ peak can introduce another layer of variance and the larger the ${T}_n$ is, the smaller the variance is. For simplicity, we only investigate the impact of the variances from the Beta distributions on performance. Here, two cases were considered; in the first case, moderate variances of the methylation degree were simulated where $\left[\boldsymbol{\alpha}, \boldsymbol{\beta} \right]$=$\left[16,2;16,4;20,10;25,10\right]$and in the second case, the variances were assumed very high and set as $\left[\boldsymbol{\alpha}, \boldsymbol{\beta} \right]=\left[8,1;4,1;1.2,1;9,10\right]$. To best mimic the real MeRIP-Seq data, $N=10000$ methylation peaks and $M=2$ replicates were simulated. Also, we let the total count ${T}_n=100$ for any methylation peak.

**Fig. 2:**
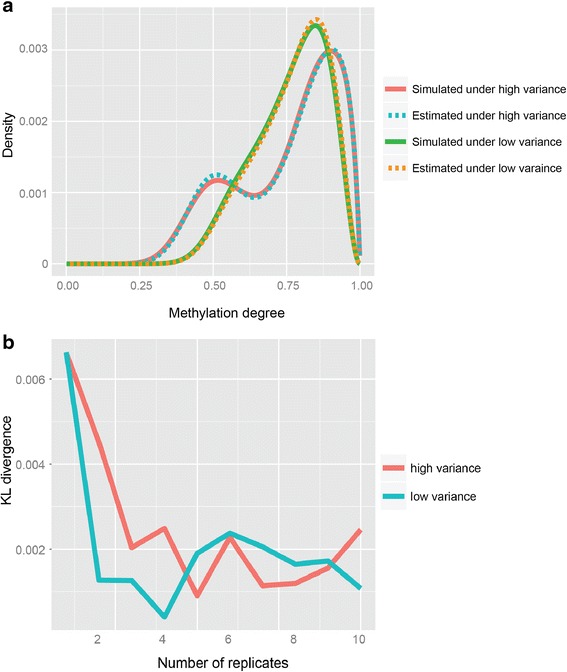
Performance evaluation on simulated m^6^A peaks. **a**. The algorithm performs well on both moderate and high variances cases. **b**. As the number of replicates increases, the performance is enhanced

The performance of the proposed algorithm in uncovering the clusters of m^6^A peak methylation degree can be evaluated by examining the goodness-of-fit of the mixture Beta distribution (15). Figure 2a demonstrates that the fitting performance for both moderate and high variance cases both cases and we can see the estimated mixture density is extremely close to the true ones, indicating a good fitting performance by the algorithm. In order to quantify the influence of the number of replicates on the fitting performance, simulated datasets with replicates varying from 1 to 10 were generated. The goodness-of-fit measured by Kullback–Leibler (KL) divergence between the estimated and the true mixture distributions was examined for different number of replicates separately. We can see from Fig. 2b that even with no replicate the fitting performance is very high with a KL divergence less 0.7 %. When there are two or more replicates, further improvement can be obtained, where the KL divergence can be reduce to as low as 0.2 %. Taken together, the results provide strong evidence to support a good fitting performance of the proposed algorithm for different reads variations.

### Evaluation on real m^6^A MeRIP-seq data

To further validate the accuracy of the proposed algorithm, we applied it to two real public available m^6^A MeRIP-seq datasets [5, 8]. One is from the mouse midbrain cells including 3 replicates, download from Gene Expression Omnibus (GEO) (accession number GSE47217) and the other dataset including 4 replicates measures transcriptome-wide m^6^A in human HeLa cells (accession number GSE46705). The datasets were pre-processed according to the HEPeak pipeline and for midbrain dataset, a total of 18162 m^6^A peaks were identified, whereas 7243 m^6^A peaks were reported in the HeLa cells both for FDR < 0.05.

**Fig. 3:**
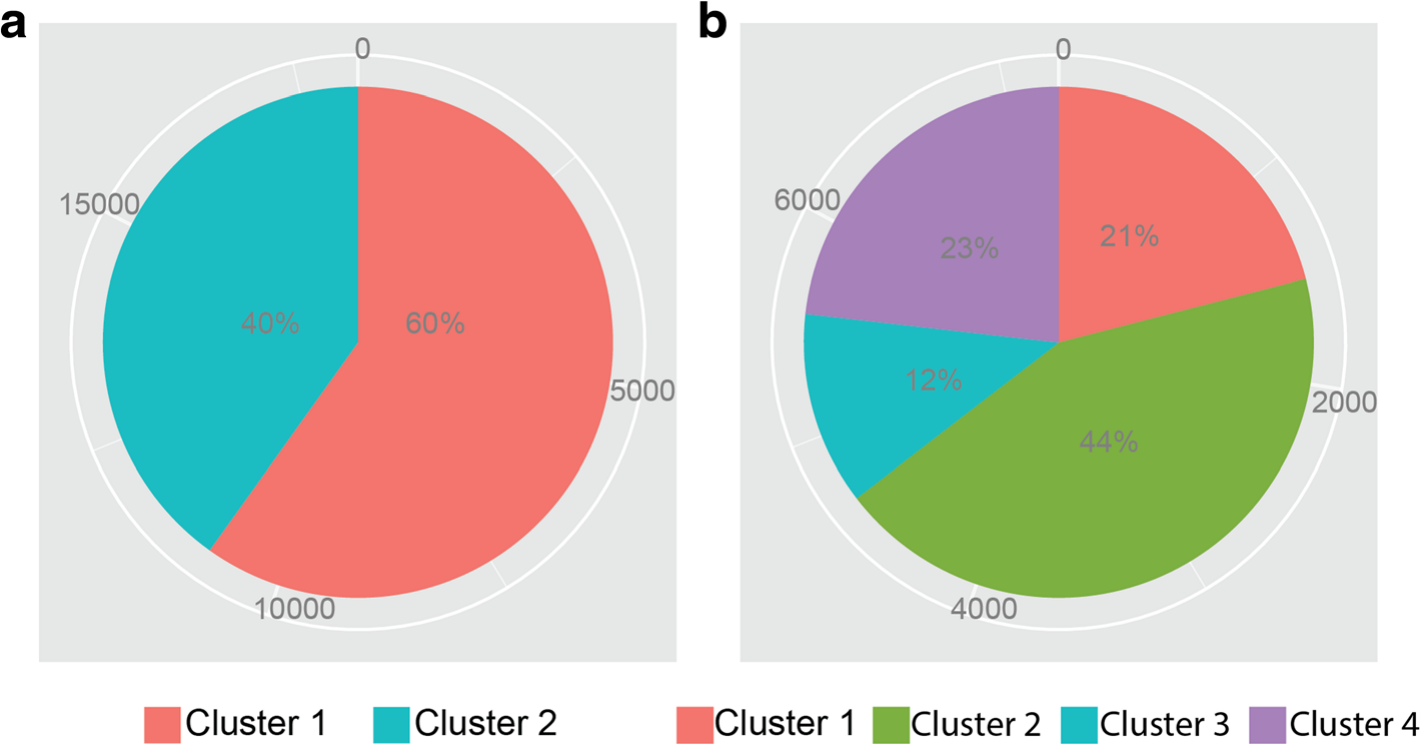
Pie chart for the proportion of peaks in each m^6^A clusters discovered in mouse midbrain and human HeLa cells. **a**. An illustration of the proportion of m^6^A peaks in each clusters in mouse midbrain cells. **b**. An illustration of the proportion of m^6^A peaks in each clusters in human HeLa cells

Next, we applied our algorithm to uncover the peak clusters in two datasets. 2 m^6^A peak clusters were determined to exist for the mouse midbrain cells (Fig. 3a), where cluster 1 contains 60 % (10875) of the peaks and cluster 2 includes the remaining 40 % (7287). In contrast, 4 different m^6^A peak clusters were discovered for HeLa cells (Fig. 3b), with the proportion of peaks as 21 % (1521) for cluster 1, 44 % (3155) for cluster 2, 12 % (886) for cluster 3, and 23 % (1681) for cluster 4, where the cluster is ranked according to a descending order of methylation degree.

**Fig. 4:**
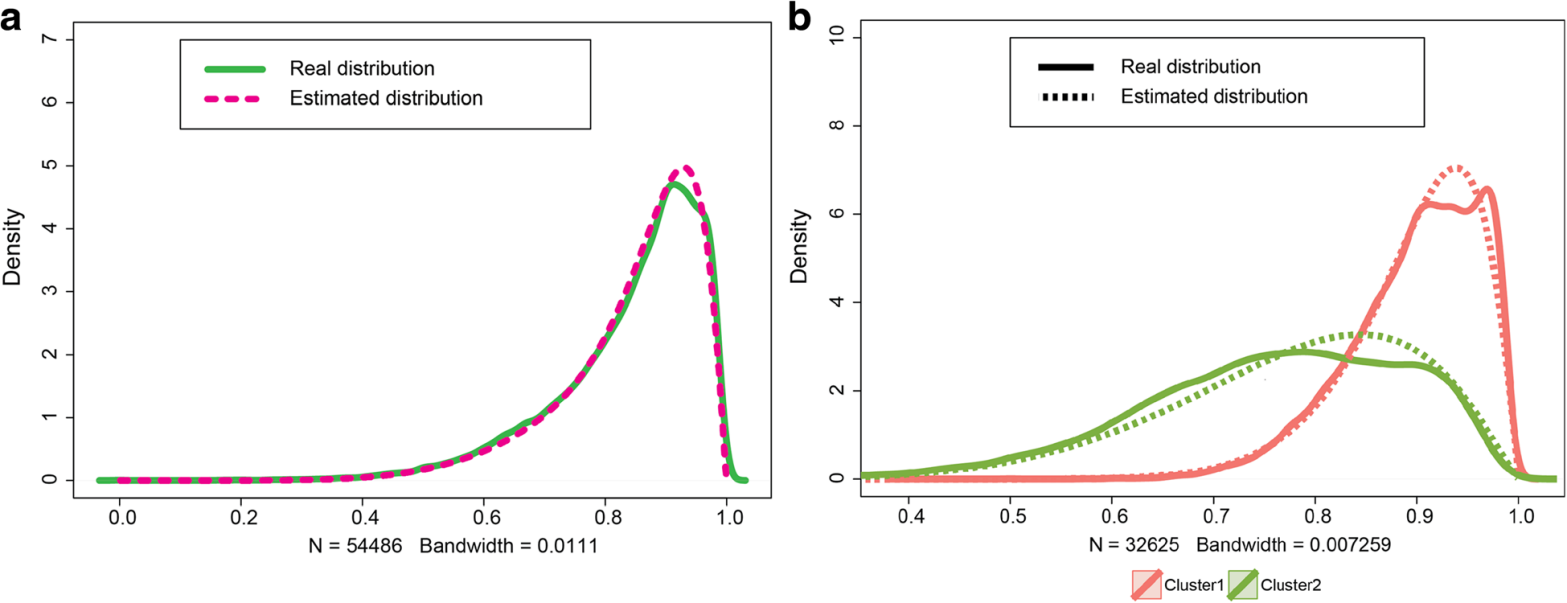
The estimated mixture density closely characterizes the real distribution of m^6^A peak in mouse midbrain cells. **a**. The estimated mixture Beta distribution versus the overall distribution of real m^6^A peaks in mouse midbrain cells. **b**. Comparison between the two estimated mixture components and the real distributions of m^6^A peaks in the corresponding cluster

**Fig. 5:**
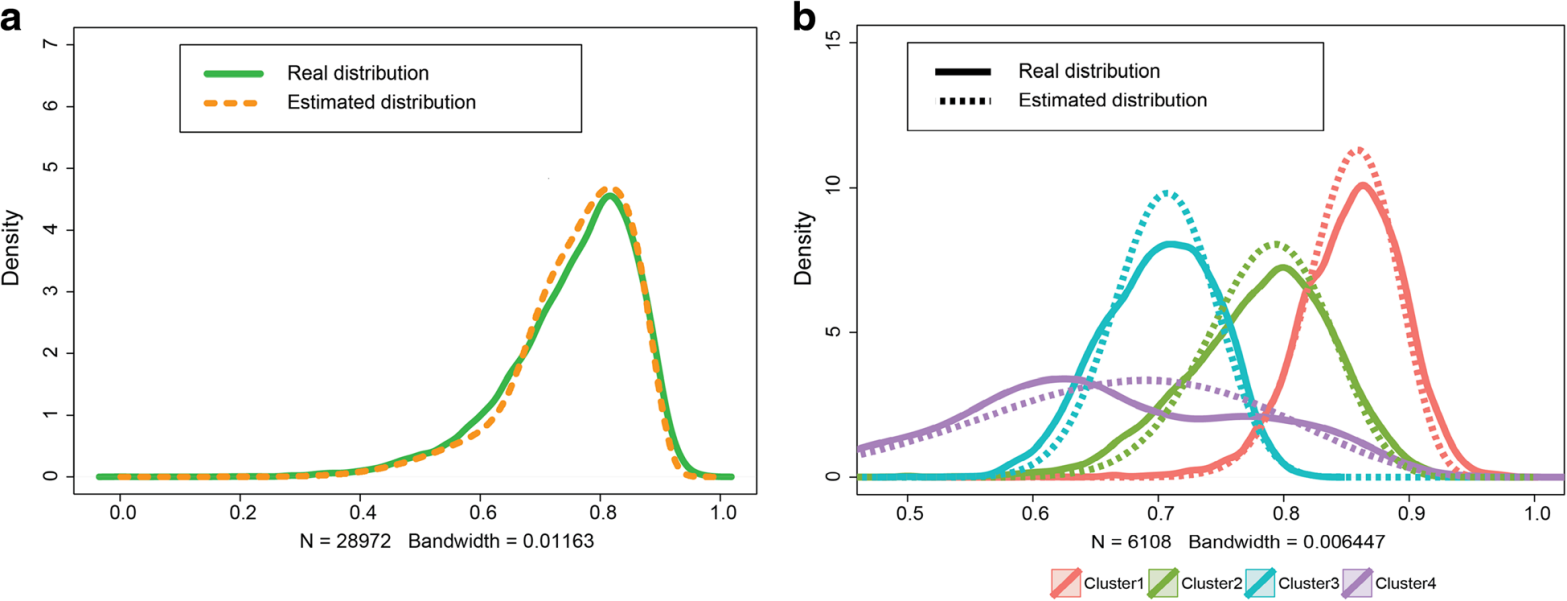
The estimated mixture density closely characterizes the real distribution of m^6^A peak in HeLa cells. **a**. The estimated density versus the overall distribution of methylation degree of m^6^A peaks in human HeLa cells. **b**. Comparison between the four estimated mixture components and the real distributions of m^6^A peaks in the corresponding cluster

To evaluate the accuracy of the proposed algorithm in characterizing the true mixture distribution of the methylation degree, the estimated density was next tested against the empirical distribution of peak methylation degrees obtained from MeRIP-Seq data. As illustrated in Figs. 4a and 5a, the estimated mixture distributions capture the real distributions of methylation degrees very well for both mouse and human MeRIP-seq datasets. We further investigated each components of the mixture. Figure 4b shows the empirical peak distributions of the two uncovered clusters in the mouse midbrain, which have distinct patterns. The fitted distributions of each cluster well captured the corresponding empirical distribution (chi-square test, *p*value: 9.2e-14 and 4.4e-4 for cluster 1 and 2). For human HeLa cells Fig. 5b, four distinct empirical distributions of peaks can be clearly seen and high fitting performance was also achieved for all four clusters (chi-square test, *p*value: 5.8e-21, 7.48e-38, 1.1e-15 and 1.2e-8 for cluster 1 to 4).

### A novel pattern of m^6^A distribution is revealed

**Fig. 6:**
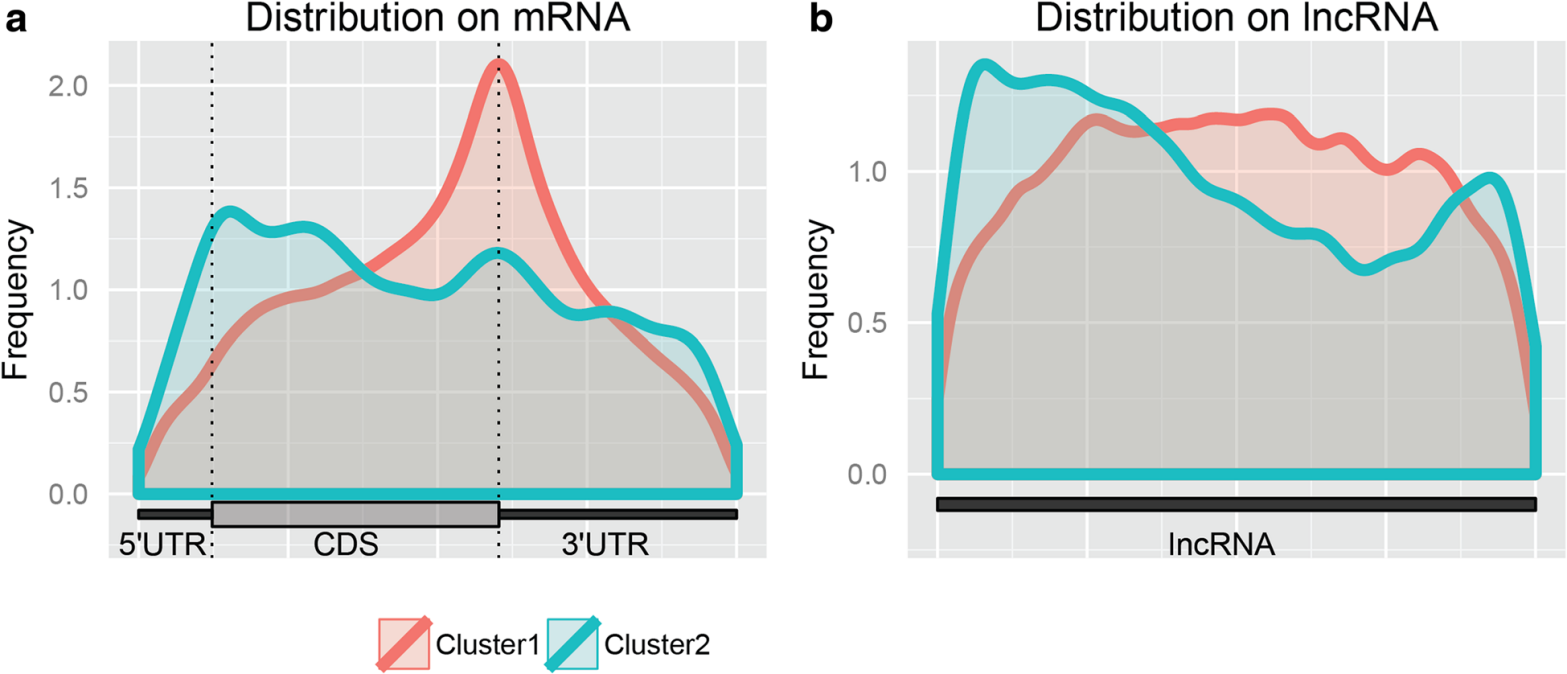
Distribution of m^6^A for different clusters in mRNA and lncRNA in mouse midbrain cells. **a**. The distribution of m^6^A peaks for different clusters in mRNA. **b**. The distribution of m^6^A peaks for different clusters in lncRNA

**Fig. 7:**
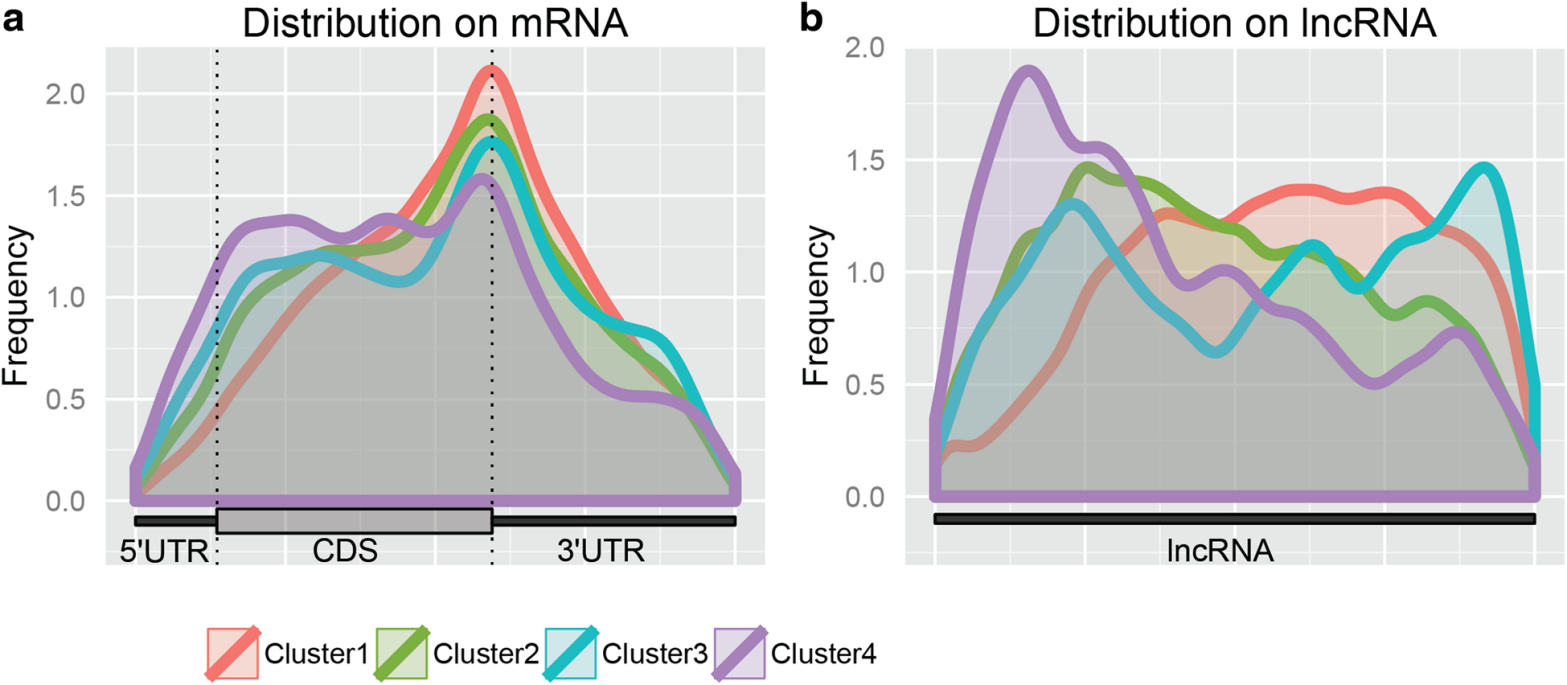
Distribution of m^6^A for different clusters in mRNA and lncRNA in human HeLa cells. **a**. The distribution of m^6^A peaks for different clusters in mRNA. **b**. The distribution of m^6^A peaks for different clusters in lncRNA

In order to gain insights into different clusters of methylation peaks, peaks in each cluster were mapped to the corresponding mRNA or lncRNA and their distribution was subsequently examined. In mouse midbrain cells, noticeable differences in the distributions of two clusters can be observed on mRNA (Fig. 6a). Peaks in cluster 1 that have higher methylation degree are highly enriched near the stop codon, a distribution similar to the general m^6^A distribution previously reported in the literature [1, 12, 13, 20], whereas those in cluster 2 that have less degree of methylation are clearly more enriched near the start codon towards the 5′ UTR. Interestingly, m^6^A peak clusters in lncRNA (Fig. 6b) also show the same pattern where the higher methylated peaks are more likely to be enriched toward its 3′UTR. This phenomenon was further supported by the results in human HeLa cells (Fig. 7a, b). We see once again that the highly methylated peaks tend to locate around the stop codon and the peaks move towards the 5′ end as their methylation degree decreases. This pattern was also verified on additional MeRIP-seq datasets (Additional files 1: Figure S1 and Additional file 2: Figure S2).

**Fig. 8:**
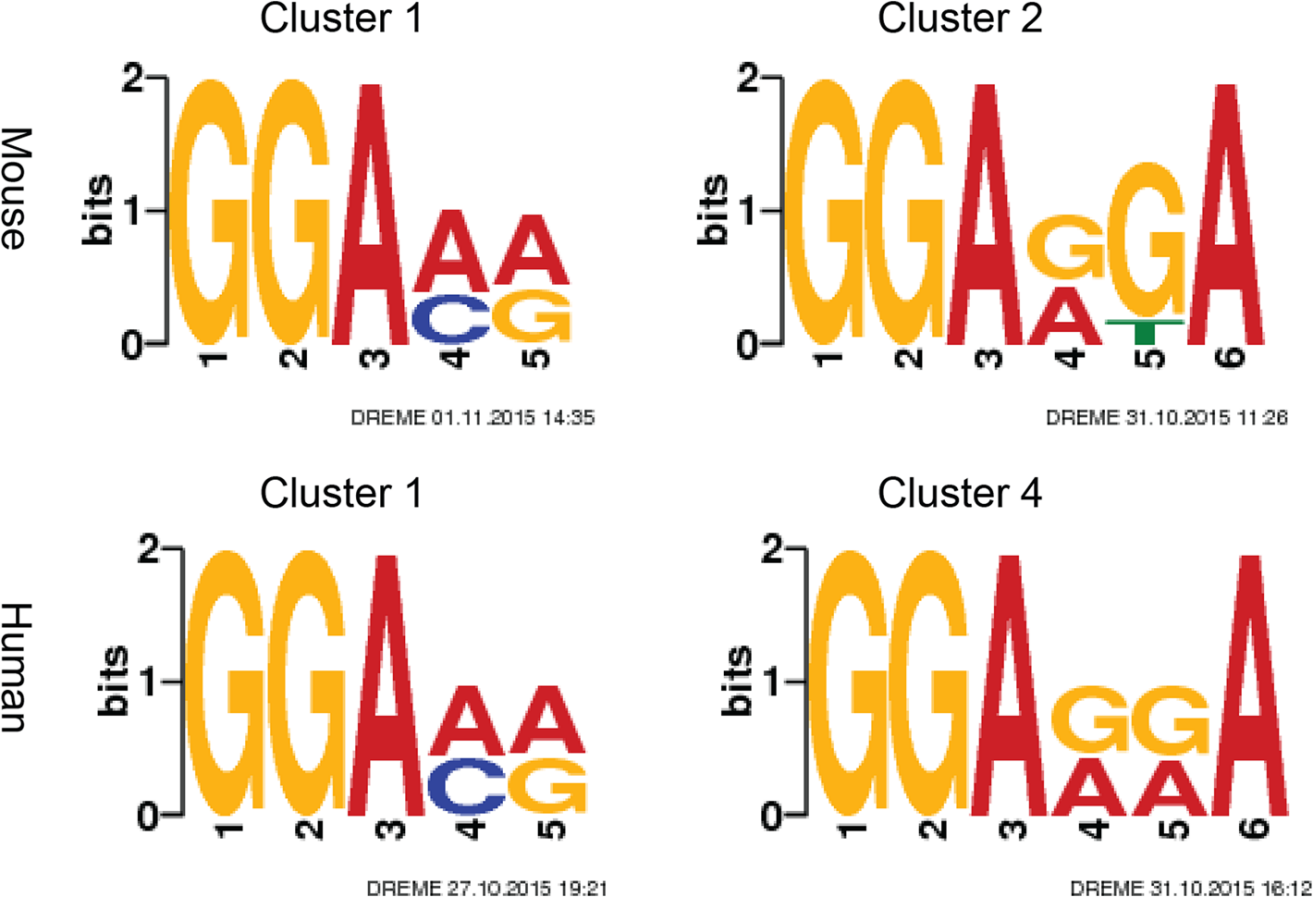
Motifs detected by DREME in human and mouse cells

To gain additional insights into these m^6^Aclusters, sequence motifs searching was performed on the sequences of the predicted m^6^A peaks for each particular cluster. The sequences of peaks were obtained by bedtools2.1 and motif search was done by using DREME [21, 22], with the shuffled sequences as the background. The most enriched consensus motifs are illustrated in the Fig. 8 and Additional file 3: Figure S3 in Additional files. Interestingly, the motifs for the highest methylated cluster in both mouse midbrain cells and human HeLa cells are found to be very similar and this similarity also exists for the lowly methylated cluster. For the highest methylated cluster, the common motif is GGAC, which has been shown by PAR-CLIP experiments as the binding motif of methyltransferase METTL14 [8]. For the lowest methylated peaks, the motif is determined as GGAGGA. This distinct motif has not been reported to be associated with any protein binding and thus requires further investigation.

## Discussion and Conclusions

In this paper, a novel graphical model based methylation peak clustering algorithm, was developed for discovering the patterns in methylation degrees of m^6^A peaks in the MeRIP-seq data. The peak cluster is modelled as the mixture Beta-binomial distribution, where the Beta distribution can model the variance of the methylation degree across sample replicates. The evaluation on both simulation and real MeRIP-seq datasets demonstrates the accuracy and robustness of our model. In addition, our algorithm successfully uncovered a unique and novel pattern for m^6^A peak cluster, providing a new lead for understanding the mechanisms and functions of m^6^A methylation.

## Abbreviations

BIC, Bayesian Information Criterion; CDS, Coding DNA sequence; EM, Expectation of maximum likelihood method; FDR, False discovery rate; MeRIP-seq, Methylated RNA Immunoprecipatation combined with RNA sequencing; UTR, Untranslated region

## Additional files

Additional MeRIP-seq datasets were further examined. One experiment was conducted by knocking out an m^6^A demethylase obesity associated protein (KO-FTO) in mouse midbrain cells. The other MeRIP-seq dataset was generated by knocking out m^6^A methyltransferase METTL14 (KO-METTL14) in human HeLa cells. Additional file 1: Figure S1. Distribution of m^6^A for different clusters in mRNA and lncRNA in KO-FTO mouse midbrain cells. A. The distribution of m^6^A peaks for different clusters in mRNA. B. The distribution of m^6^A peaks for different clusters in lncRNA. (PNG 114 kb) Additional file 2: Figure S2. Distribution of m^6^A for different clusters in mRNA and lncRNA in KO-METTL14 human HeLa cells. (PNG 173 kb) Additional file 3: Figure S3. Motifs for Cluster 2 and 3 detected by DREME in human HeLa cells. (PNG 21 kb)

## References

[1] Pan T (2013). N6-methyl-adenosine modification in messenger and long non-coding RNA. Trends Biochem Sci.

[2] Liu J, Jia G. Methylation Modifications in Eukaryotic Messenger RNA. J Genet Genomics 2014;41(1);21–33. 10.1016/j.jgg.2013.10.002 24480744

[3] Desrosiers R, Friderici K, Rottman F (1974). Identification of methylated nucleosides in messenger RNA from Novikoff hepatoma cells. Proc Natl Acad Sci U S A.

[4] He C (2010). Grand challenge commentary: RNA epigenetics?. Nat Chem Biol.

[5] Hess ME, Hess S, Meyer KD, Verhagen LA, Koch L, Bronneke HS, Dietrich MO, Jordan SD, Saletore Y, Elemento O (2013). The fat mass and obesity associated gene (Fto) regulates activity of the dopaminergic midbrain circuitry. Nat Neurosci.

[6] Zheng G, Dahl JA, Niu Y, Fedorcsak P, Huang CM, Li CJ, Vågbø CB, Shi Y, Wang WL, Song SH (2013). ALKBH5 Is a Mammalian RNA Demethylase that Impacts RNA Metabolism and Mouse Fertility. Mol Cell.

[7] Wang Y, Li Y, Toth JI, Petroski MD, Zhang Z, Zhao JC (2014). N6-methyladenosine modification destabilizes developmental regulators in embryonic stem cells. Nat Cell Biol.

[8] Liu J, Yue Y, Han D, Wang X, Fu Y, Zhang L, Jia G, Yu M, Lu Z, Deng X (2014). A METTL3-METTL14 complex mediates mammalian nuclear RNA N6-adenosine methylation. Nat Chem Biol.

[9] Meyer KD, Jaffrey SR (2014). The dynamic epitranscriptome: N6-methyladenosine and gene expression control. Nat Rev Mol Cell Biol.

[10] Jia G, Fu Y, He C (2013). Reversible RNA adenosine methylation in biological regulation. Trends Genet.

[11] Hussain S, Aleksic J, Blanco S, Dietmann S, Frye M (2013). Characterizing 5-methylcytosine in the mammalian epitranscriptome. Genome Biol.

[12] Meyer KD, Saletore Y, Zumbo P, Elemento O, Mason CE, Jaffrey SR (2012). Comprehensive analysis of mRNA methylation reveals enrichment in 3′ UTRs and near stop codons. Cell.

[13] Dominissini D, Moshitch-Moshkovitz S, Schwartz S, Salmon-Divon M, Ungar L, Osenberg S, Cesarkas K, Jacob-Hirsch J, Amariglio N, Kupiec M (2012). Topology of the human and mouse m6A RNA methylomes revealed by m6A-seq. Nature.

[14] Wang Z, Gerstein M, Snyder M (2009). RNA-Seq: a revolutionary tool for transcriptomics. Nat Rev Genet.

[15] Meng J, Lu Z, Liu H, Zhang L, Zhang S, Chen Y, Rao MK, Huang Y. A protocol for RNA methylation differential analysis with MeRIP-Seq data and exomePeak R/Bioconductor package. Methods 2014 10.1016/j.ymeth.2014.06.008 PMC4194139 24979058

[16] Meng J, Cui X, Rao MK, Chen Y, Huang Y (2013). Exome-based analysis for RNA epigenome sequencing data. Bioinformatics.

[17] Xiaodong Cui JM, Manjeet K. Rao, Yidong Chen, Yufei Huang. HEP: An HMM-based Exome Peak-finding Package for RNA Epigenome Sequencing Data. BMC Genomics 2014 10.1186/1471-2164-16-S4-S2 PMC4416174 25917296

[18] Posada D, Buckley TR (2004). Model selection and model averaging in phylogenetics: advantages of Akaike information criterion and Bayesian approaches over likelihood ratio tests. Syst Biol.

[19] Lindstrom MJ, Bates DM (1988). Newton—Raphson and EM algorithms for linear mixed-effects models for repeated-measures data. J Am Stat Assoc.

[20] Schwartz S, Agarwala SD, Mumbach MR, Jovanovic M, Mertins P, Shishkin A, Tabach Y, Mikkelsen TS, Satija R, Ruvkun G. High-Resolution Mapping Reveals a Conserved, Widespread, Dynamic mRNA Methylation Program in Yeast Meiosis. Cell 2013 10.1016/j.cell.2013.10.047 PMC3956118 24269006

[21] Bailey TL (2011). DREME: motif discovery in transcription factor ChIP-seq data. Bioinformatics.

[22] Machanick P, Bailey TL (2011). MEME-ChIP: motif analysis of large DNA datasets. Bioinformatics.

