# Supplementary figures and images for: A hierarchical model for clustering m6A methylation peaks in MeRIP-seq data
Source: BMC Genomics. 2016 Aug 22;17(Suppl 7):520. doi: 10.1186/s12864-016-2913-x (PMC5001242; doi:10.1186/s12864-016-2913-x)

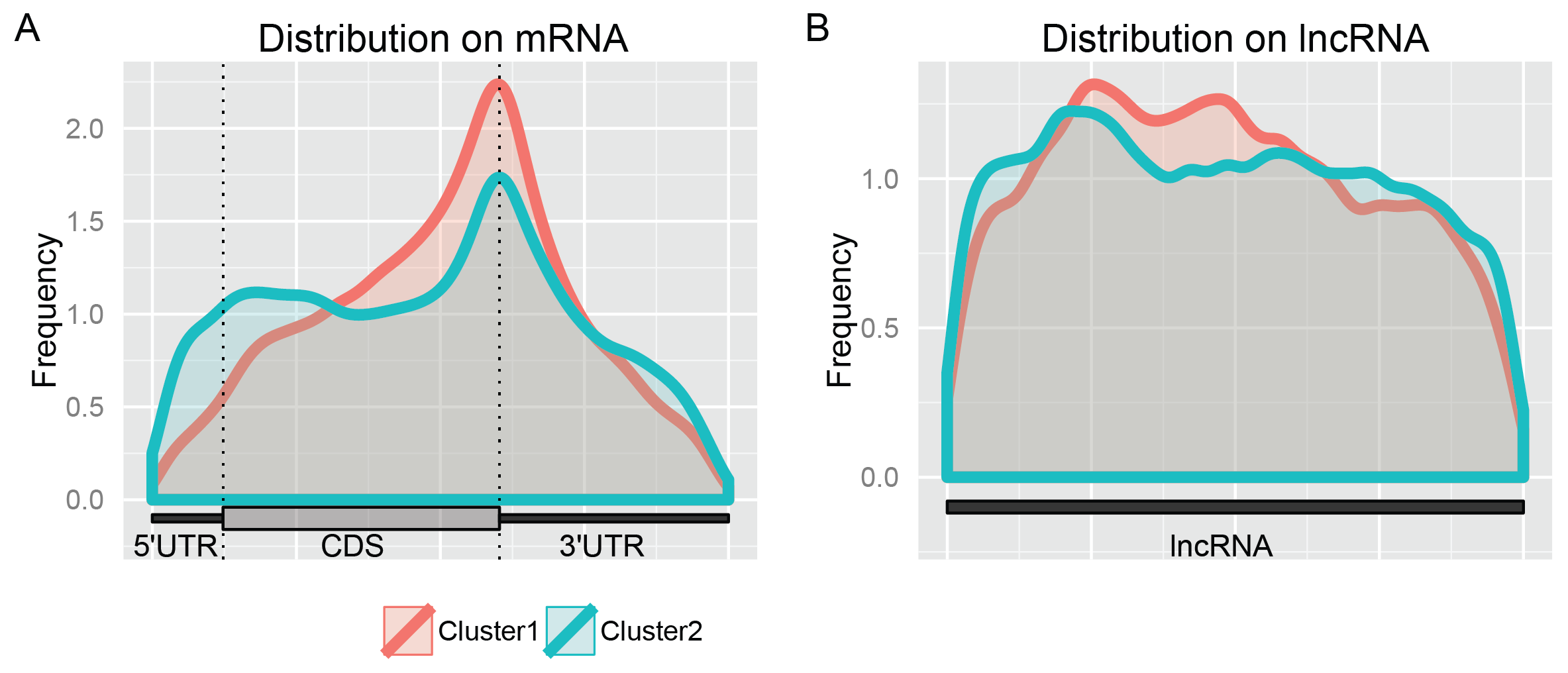

Supplement: Additional file 1: Figure S1. — Distribution of m6A for different clusters in mRNA and lncRNA in KO-FTO mouse midbrain cells. A. The distribution of m6A peaks for different clusters in mRNA. B. The distribution of m6A peaks for different clusters in lncRNA. (PNG 114 kb) [file 12864_2016_2913_MOESM1_ESM.png]

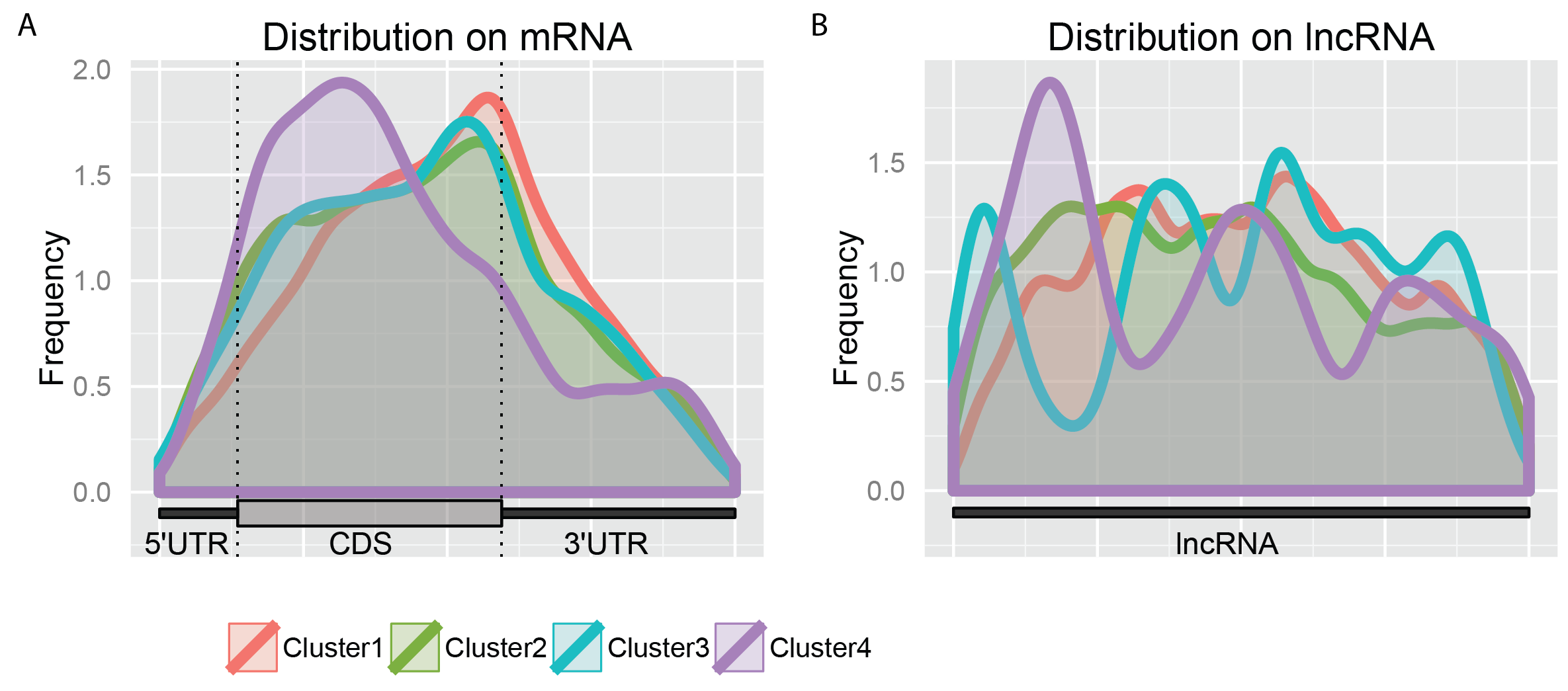

Supplement: Additional file 2: Figure S2. — Distribution of m6A for different clusters in mRNA and lncRNA in KO-METTL14 human HeLa cells. (PNG 173 kb) [file 12864_2016_2913_MOESM2_ESM.png]

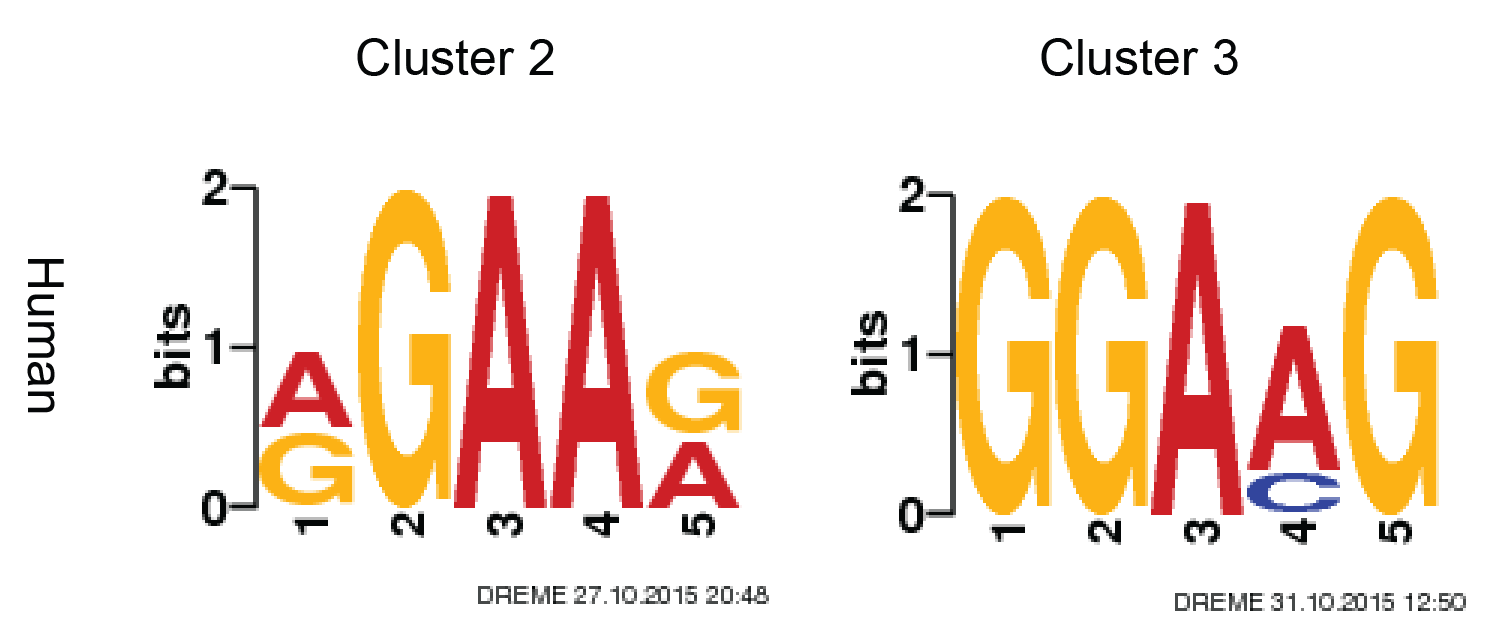

Supplement: Additional file 3: Figure S3. — Motifs for Cluster 2 and 3 detected by DREME in human HeLa cells. (PNG 21 kb) [file 12864_2016_2913_MOESM3_ESM.png]
